# A multimodal MRI radiomics sonata for differentiating small renal masses: a two-center retrospective study

**DOI:** 10.1016/j.clinsp.2026.101212

**Published:** 2026-10-16

**Authors:** Chao Fang, Li Tian, Ming Gao, Xiaohui Duan, Qi Liu, Zixin Xu, Junjiong Zheng, Yushu Chen, Ming Huang, Xu Chen, Wei Jiang, Jianqiu Kong, Wen Dong

**Affiliations:** a Department of Urology, Guangdong Provincial Key Laboratory of Malignant Tumor Epigenetics and Gene Regulation, Sun Yat-sen Memorial Hospital, Sun Yat-sen University, Guangzhou, China; b Department of Medical Imaging, State Key Laboratory of Oncology in South China, Guangdong Provincial Clinical Research Center for Cancer, Collaborative Innovation Center for Cancer Medicine, Sun Yat-Sen University Cancer Center, Guangzhou, Guangdong, China; c Department of Radiology, Sun Yat-sen Memorial Hospital, Sun Yat-sen University, Guangzhou, Guangdong, China

**Keywords:** Diagnosis, Magnetic resonance imaging, Radiomics, Small Renal Masses

## Abstract

**Background:**

Accurately distinguishing benign from malignant small renal masses (SRMs) is crucial to avoid unnecessary surgeries, yet reliable preoperative diagnostic tools remain limited.

**Methods:**

A total of 189 patients with SRMs from two hospitals (2018–2022) were enrolled. Clinical data and MRI features were analyzed by two radiologists. Radiomics features were automatically extracted from manually segmented ROIs using dedicated software. Significant features were selected via univariate analysis, multivariate regression, and Lasso to develop three models: radiomics, clinical, and combined nomogram. Diagnostic performance was evaluated using the Area Under the receiver operating characteristic Curve (AUC).

**Results:**

The radiomics-clinical nomogram was constructed using T2WI signal intensity (p < 0.001), pseudocapsule (p = 0.001), and radiomics score (Rad-score, p = 0.001) via multivariate logistic regression. In the training set, the nomogram achieved an AUC of 0.962 (95% CI: 0.931–0.993), outperforming both the radiomics model (AUC = 0.918, 95% CI: 0.862–0.974) and the clinical model (AUC = 0.856, 95% CI: 0.780–0.933). Favorable performance was observed in the internal validation set (nomogram: AUC = 0.981, 95% CI: 0.944–1.000; radiomics model: AUC = 0.912, 95% CI: 0.750–1.000; clinical model: AUC = 0.844, 95% CI: 0.578–1.000) and external validation set (nomogram: AUC = 0.971, 95% CI: 0.886–1.000; radiomics model: AUC = 0.928, 95% CI: 0.841–0.989; clinical model: AUC = 0.916,95% CI: 0.760–0.976).

**Conclusion:**

All three models enable effective and non-invasive preoperative differentiation of benign from malignant SRMs, with the radiomics-clinical nomogram exhibiting the highest diagnostic accuracy, supporting its potential as a valuable clinical tool.

## Highlights

• Combining traditional imaging and radiomics to diagnose small renal tumor nature.
• T2WI, pseudocapsule, and Rad-score predict malignancy in SRMs independently.
• This non-invsive tool may reduce unnecessry surgery and guide SRM managemen

## Introduction

According to global cancer statistics from 2022, Renal Cell Carcinoma (RCC) accounts for approximately 2.2% of all newly diagnosed malignant tumors, ranking third among urological malignancies. Additionally, RCC-related deaths contribute to about 1.6% of all cancer-associated mortalities, underscoring its clinical significance.^1^ Historically, RCC was commonly identified when patients presented with one of the classic triad of symptoms ‒ hematuria, flank pain, or an abdominal mass. However, this presentation has become increasingly rare in recent years, largely due to advancements in imaging technologies.^2^ With the widespread adoption of abdominal imaging modalities, the detection rate of renal masses has risen significantly, with a substantial proportion being Small Renal Masses (SRMs).^3^ Defined as lesions measuring ≤4 cm in maximum diameter on abdominal imaging,^4^ SRMs exhibit a heterogeneous pathological spectrum: approximately 80% of resected SRMs are malignant, while 20% are benign.^5^^,^^6^ This discrepancy implies that a considerable number of patients may undergo unnecessary surgical interventions, highlighting the critical need for accurate preoperative characterization of SRMs, a major challenge and active research focus in contemporary clinical practice.

Tissue biopsy serves as the gold standard for the preoperative diagnosis in most cancer, such as breast and prostate cancer. however, in RCC, renal mass biopsy is limited by factors including sampling error and potential complications, restricting its use to an adjunctive role.^7^ In addition, unlike hepatocellular carcinoma, there are no reliable serum tumor markers validated for auxiliary diagnosis of RCC. Consequently, imaging remains the cornerstone of preoperative RCC assessment. Enhanced Computed Tomography (CT) is currently the most widely used imaging tool; yet its diagnostic performance for SRMs is constrained by inherent limitations in soft tissue resolution.^8^ Magnetic Resonance Imaging (MRI), with its multiple sequences and superior ability to delineate tumor characteristics compared to CT,^9^ has been explored in numerous studies for differentiating benign and malignant SRMs. However, these efforts have yielded suboptimal results, with substantial overlap in qualitative imaging features .^10^, ^11^, ^12^ The clear cell Likelihood Score (ccLS) has also been applied to SRM diagnosis .^13^ However, its primary utility lies in distinguishing clear cell RCC from other renal tumor subtypes, rather than achieving true benign-malignant differentiation. This limitation makes ccLS insufficient to meet the clinical needs for differentiating benign and malignant SRMs.

In the recent years, radiomics has emerged as a promising technology, enabling high-throughput, automated quantification of phenotypic characteristics from medical images. This approach has shown potential in tumor classification, prognosis prediction, and treatment response assessment .^14^^,^^15^ While several studies have demonstrated the utility of CT-based radiomics for characterizing SRM pathology,^16^, ^17^, ^18^, ^19^ research on MRI-based radiomics in this context remains limited, with only a few studies exploring this area.^20^

Against this backdrop, the present study aimed to develop and validate a nomogram integrating clinical data, conventional MRI features, and MRI-based radiomics features to preoperatively distinguish between benign and malignant SRMs. The model integrates tumor-region-specific radiomic features with clinical covariates to minimize confounding effects from background renal tissue, a design that addresses the limitations of whole-kidney radiomics models reported in recent studies. By enhancing diagnostic accuracy, this tool seeks to reduce unnecessary surgical interventions and optimize clinical decision-making.

## Materials and methods

### Patients

A total of 189 patients with pathologically confirmed Small Renal Masses (SRMs) who underwent preoperative multiphasic contrast-enhanced Magnetic Resonance Imaging (MRI) between 2018 and 2022 were enrolled in this study. Patients who received active surveillance instead of surgical intervention were excluded due to the lack of definitive pathological diagnosis, which is a prerequisite for radiomic model development and validation. Among them, 147 patients were recruited from Sun Yat-sen Memorial Hospital, and the remaining 42 patients were from Sun Yat-Sen University Cancer Center. The inclusion criteria were as follows: 1) Pathologically confirmed renal masses; 2) Complete multiphasic contrast-enhanced MRI image; 3) Renal masses have a maximum size of ≤ 4 cm on MRI. The exclusion criteria were: 1) Patients who had received other anticancer treatments, such as targeted therapy or immunotherapy before surgery; 2) Poor or damaged image quality because of image artifacts and overlaps; 3) Tumors with no solid component.

This study was approved by the institutional ethics committee of both participating hospitals, and the requirement for individual informed consent was waived due to the retrospective nature of the study. This study followed the STARD guidelines.

### MRI imaging

MRI scans were performed using either 1.5 Tesla or 3 Tesla scanners across multiple centers. Images used for radiomic analysis included axial or coronal non-fat-suppressed T2-Weighted Imaging (T2WI), fat-suppressed T2WI, and Contrast-Enhanced T1-Weighted Imaging (CE-T1WI). For non-fat-suppressed T2WI, the Repetition Time (TR) ranged from 3000–4000 ms, Echo Time (TE) from 80–100 ms, slice thickness of 3 mm, and matrix size of 256×256. For CE-T1WI, TR was 400–600 ms, TE 10–20 ms, slice thickness 3 mm, matrix size 256×256. CE-T1WI was acquired 50–60 seconds after intravenous administration of a Gadolinium-based contrast agent (Gd-DTPA) via the antecubital vein at a dose of 0.1 mmol/kg and an injection rate of 3.0 mL/s.

### Evaluation of conventional MRI features

Two experienced radiologists independently reviewed and assessed the following MRI imaging features: 1) Maximum diameter of the renal mass (measured on axial MRI); 2) Signal intensity on fat-suppressed T2WI (classified as low, isointense, or high); 3) Signal heterogeneity on fat-suppressed T2WI (homogeneous, minimally heterogeneous, or heterogeneous); 4) Presence or absence of a pseudocapsule; 5) Presence or absence of bulk fat; 6) Degree of contrast enhancement (low, moderate, or high); 7) Enhancement heterogeneity (homogeneous, minimally heterogeneous, or heterogeneous) .^21^ Inter-rater agreement for these categorical variables was evaluated using the kappa statistic. Discrepancies between the two radiologists were resolved through consensus by joint review.

### Region of interest (ROI) delineation and radiomic feature extraction

Original DICOM-format MRI images (with preserved size and resolution) were retrieved for analysis. For each case, manual segmentation of the renal mass was performed using 3D Slicer software (version 5.2.1) .^22^

A trained physician proficient in 3D Slicer delineated ROIs on both non‑fat‑suppressed T2WI and CE‑T1WI according to the following standardized protocol, which clearly defines that segmentation should be performed along the inner tumor boundary with a 1-mm margin. Anatomical landmarks, including the renal capsule and the tumor-normal tissue interface, were consistently applied to ensure uniform ROI delineation across all cases.

After image preprocessing (resampling to a voxel size of 1 × 1 × 1 mm and grayscale discretization with a bandwidth of 25), radiomic features were automatically extracted using the Pyradiomics plugin. These features included 744 wavelet features, 75 texture features, 18 first-order features, and 14 shape features, resulting in a total of 851 features extracted from an MRI sequence. To assess the reproducibility of feature extraction, 30 cases were randomly selected, and the same physician re-delineated ROIs and re-extracted features two weeks later. The Intraclass Correlation Coefficient (ICC) was used for validation, with an ICC > 0.75 indicating good reliability.

### Model construction and performance comparison

The 147 patients from Sun Yat-sen Memorial Hospital were randomly assigned to a training cohort and an internal validation cohort at a ratio of 3:1. The 42 patients from Sun Yat-Sen University Cancer Center served as the external validation cohort. Using univariate analysis and multivariate logistic regression, we identified statistically significant clinical factors and established the clinical model. To evaluate potential multicollinearity among the independent variables, Variance Inflation Factors (VIFs) were calculated. A threshold of VIF > 5 was adopted to indicate the presence of notable multicollinearity. After performing *z*-score normalization, the LASSO regression was applied to pick radiomic features and tenfold cross-validation procedure was used to select an appropriate λ for radiomic model construction. The radiomics score (Rad-score) was calculated by weighting picked features according to their coefficients in the model. In the training cohort, multivariate logistic regression, including Rad-score and significant clinical factors, was completed to generate the radiomics nomogram. The calibration curve was generated based on the Hosmer-Lemeshow test to appraise the model's goodness of fit. The performance of the three models across the three cohorts was evaluated using Receiver Operating Characteristic (ROC) curve analysis and AUC. The DeLong test was employed to assess statistical differences between the AUCs of the models. The net clinical benefit of three models in distinguishing malignant and benign SRMs was appraised through Decision Curve Analysis (DCA).

### Statistical analysis

All statistical analyzes were completed in R software, version 4.3.0. Chi-Square test and *t*-test were applied to compare qualitative data and quantitative data respectively. The univariate and multivariate analysis were completed with the “autoReg” package. VIFs were calculated using the 'car' package to assess multicollinearity among the independent variables. Using the “rms” package, the clinical model, calibration curves, and radiomics nomogram were constructed. The LASSO regression and the Hosmer-Lemeshow test were completed using the “glmnet” package and “ResourceSelection” package respectively. The ROC curves DCA were completed using the “pROC” package and “rmda” package respectively. Statistical significance was defined as a p-value < 0.05.

## Results

### Patient characteristics and clinical model building

Table 1 shows the clinical data and MRI imaging features of the 189 patients. The median age of the enrolled patients was 53.74±12.7 years, with males accounting for 64% of the study population. The average tumor size was 2.50±0.82 cm, and 57.1% of the tumors were located in the left kidney. Benign lesions constituted 22.8% of all included small renal masses. Regarding the assessment of MRI features, inter-rater agreement was excellent, with kappa values for all evaluated features exceeding 0.6 (Supplementary Fig. 1). Within the training cohort, univariate analysis showed that sex (p < 0.001), T2WI signal intensity (p < 0.001), T2WI signal heterogeneity (p < 0.001), pseudocapsule (p < 0.001), contrast enhancement (p < 0.001), and enhancement heterogeneity (p < 0.001) were statistically significant. Multivariate logistic regression analysis showed that only high signal intensity on fat-suppressed T2WI (p = 0.006) and presence of pseudocapsule (p = 0.02) were independent risk factors of malignancy (Table 2). The VIF values of all clinical variables were less than 5, indicating that no significant multicollinearity existed between them.

**Table 1:** Clinical baseline data of patients.

Characteristics	ALL	Training cohort (n = 110)	Internal validation cohort (n = 37)	External test cohort (n = 42)	p-value
Age (years)	53.74±12.70	52.85 ± 13.02	55.49±12.48	54.52±12.19	0.706
Gender					0.836
Male	121 (64.0%)	68 (61.8%)	26 (70.3%)	27 (64.3%)	
Female	68 (36.0%)	42 (38.2%)	11 (29.7%)	15 (35.75)	
Location					0.729
Left	108 (57.1%)	64 (58.2%)	23 (62.2%)	21 (50.0%)	
Right	81 (42.9%)	46 (41.8%)	14 (37.8%)	21 (50.0)	
Tumor Size(cm)	2.50±0.82	2.61±0.82	2.40±0.81	2.32±0.82	0.228
T2W Signal Intensity					0.801
Low	36 (19.0%)	22 (20.0%)	5 (13.5%)	9 (21.4%)	
Isointense	38 (20.2%)	20 (18.2%)	8 (21.6%)	10 (23.8%)	
High	115 (60.8%)	68 (61.8%)	24 (64.9%)	23 (54.8%)	
T2W Signal Heterogeneity					0.994
Homogeneous	50 (26.5%)	28 (25.5%)	8 (21.6%)	14 (33.3%)	
Minimally heterogeneous	73 (38.6%)	46 (41.8%)	17 (45.9%)	10 (23.8%)	
Heterogeneous	66 (34.9%)	36 (32.7%)	12 (32.4%)	28 (42.9%)	
Pseudocapsule					0.014
Present	126 (66.7%)	76 (69.1%)	30 (81.1%)	20 (47.6%)	
Absent	63 (33.3%)	34 (30.9%)	7 (18.9%)	22 (52.4%)	
Bulk Fat					0.908
Present	14 (7.4%)	9 (8.2%)	3 (8.1%)	2 (4.8%)	
Absent	175 (92.6%)	101 (91.8%)	34 (91.9%)	40 (95.2%)	
Contrast Enhancement					0.081
Low	19 (10.1%)	10 (9.1%)	3 (8.1%)	6 (14.3%)	
Moderate	47 (24.9%)	23 (20.9%)	8 (21.6%)	16 (38.1%)	
High	123 (65.1%)	77 (70.0%)	26 (70.3%)	20 (47.6)	
Enhancement Heterogeneity					0.713
Homogeneous	36 (19.1%)	20 (18.2%)	5 (13.5%)	11 (26.2%)	
Minimally heterogeneous	73 (38.6%)	45 (40.9%)	14 (37.8%)	14 (33.3%)	
Heterogeneous	80 (42.3%)	45 (40.9%)	18 (48.6%)	17 (40.5%)	
Pathological Type					
Clear Cell RCC	117 (61.9)	67 (60.9%)	24 (64.9%)	26 (61.9%)	0.873
Papillary RCC	13 (6.9%)	8 (7.3%)	3 (8.1%)	2 (4.8%)	
Chromophobe RCC	10 (5.3%)	6 (5.5%)	3 (8.1%)	1 (2.4%)	
Other RCC	6 (3.2%)	4 (3.6%)	2 (5.4%)	0	
Angiomyolipoma	36 (19.0%)	20 (18.2%)	5 (13.5%)	11 (26.1%)	
Fat-poor Angiomyolipoma	22 (11.6%)	11 (10.0%)	2 (5.4%)	9 (21.4%)	
Oncocytoma	7 (3.7%)	5 (4.5%)	0	2 (4.8%)	
Tumor nature					
Malignant	146 (77.2%)	85 (77.3%)	32 (86.5%)	29 (69.0%)	0.335
Benign	43 (22.8%)	25 (22.7%)	5 (13.5%)	13 (31.0%)	

RCC, Renal Cell Carcinoma.

**Table 2:** Univariate and multivariate logistic regression analysis in training cohort.

Factors	Univariate analysis			Multivariate analysis		
	OR	95% CI	p-value	OR	95%CI	p-value
Age (years)	1.02	0.99‒1.06	0.185			
Gender (Female vs. Male)	0.20	0.07‒0.51	<0.001			
Location (Left vs. Right)	0.89	0.36‒2.19	0.801			
Tumor Size (cm)	1.02	0.59‒1.76	0.952			
T2WI Signal Intensity						
Isointense vs. Low	15.11	3.60‒84.13	<0.001	64.69	4.19–998.93	<0.01
High vs. Low	27.56	8.38‒106.40	<0.001	42.62	5.20–349.40	<0.001
T2WI Signal Heterogeneity						
Minimally heterogeneous vs. Homogeneous	2.13	0.79‒5.84	0.138			
Heterogeneous vs. Homogeneous	26.25	4.59–499.20	<0.001			
Pseudocapsule (Present vs. Absent)	14.78	5.05‒43.25	<0.001	23.87	3.66–155.74	<0.001
Bulk Fat (Absent vs. Present)	0.00	0.00‒Inf	0.988			
Contrast Enhancement						
Moderate vs. Low	1.09	0.241–4.95	0.909			
High vs. Low	7.56	1.80–32.65	<0.01			
Enhancement Heterogeneity						
Minimally heterogeneous vs. Homogeneous	4.28	1.41–13.67	<0.05			
Heterogeneous vs. Homogeneous	12.53	3.468‒54.34	<0.001			
Rad-score	5.50	2.79‒12.65	<0.001	5.18	1.93‒13.94	0.001

OR, Odd Ratio; CI, Confidence Interval.

### Study workflow and patient distribution

The overall workflow of the study, encompassing patient enrollment, radiomic feature extraction, model development (clinical model, radiomics model, and radiomics nomogram), and multi-cohort validation, is systematically illustrated in Fig. 1. A total of 189 patients with Small Renal Masses (SRMs) were included: 147 from Sun Yat-sen Memorial Hospital, randomly allocated into a training cohort (n = 110) and an internal validation cohort (n = 37) at a 3:1 ratio, and 42 from Sun Yat-Sen University Cancer Center as the external validation cohort.

**Fig. 1:**
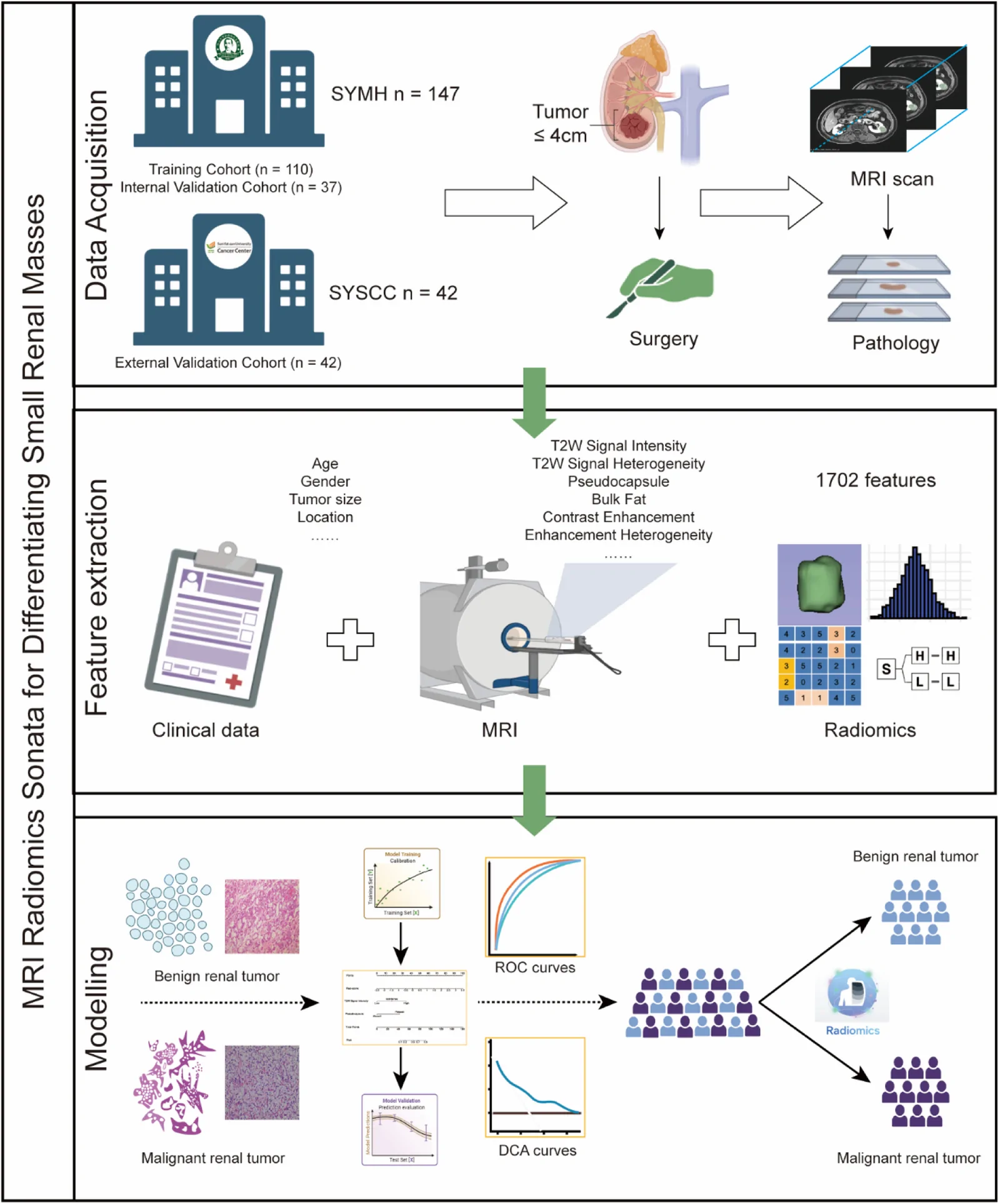
Overall research flowchart.

### Radiomics feature selection and rad-score calculation

For radiomics feature selection, the Intraclass Correlation Coefficient (ICC) was calculated for all 1702 initially extracted features, with results detailed in Supplementary Table 1 and Supplementary Figure 2. A total of 1665 features (97.8%) showed good reproducibility (ICC > 0.75) and were retained. Specifically, for the 22 radiomic features ultimately selected for constructing the model, the inter-operator ICC values ranged from 0.970 to 0.999, indicating excellent inter-operator consistency. After *z*-score normalization, LASSO regression was applied for feature selection: the process of optimal penalty parameter (λ) determination via tenfold cross-validation and feature coefficient trajectories are visualized in Fig. 2A and 2B, respectively, resulting in 22 selected features (Fig. 2C and Supplementary Table 2). The Rad-score was computed using the formula: Rad−score=2.392+7.367×A+2.128×B+1.838×C+1.047×D+0.627×E+0.332×F+0.037×G+0.003×H−0.010×I−0.013×J−0.018×K−0.020×L−0.046×M−0.122×N−0.187×O−0.346×P−0.386×Q−0.762×R−0.817×S−1.850×T−2.850×U−7.202×V.

**Fig. 2:**
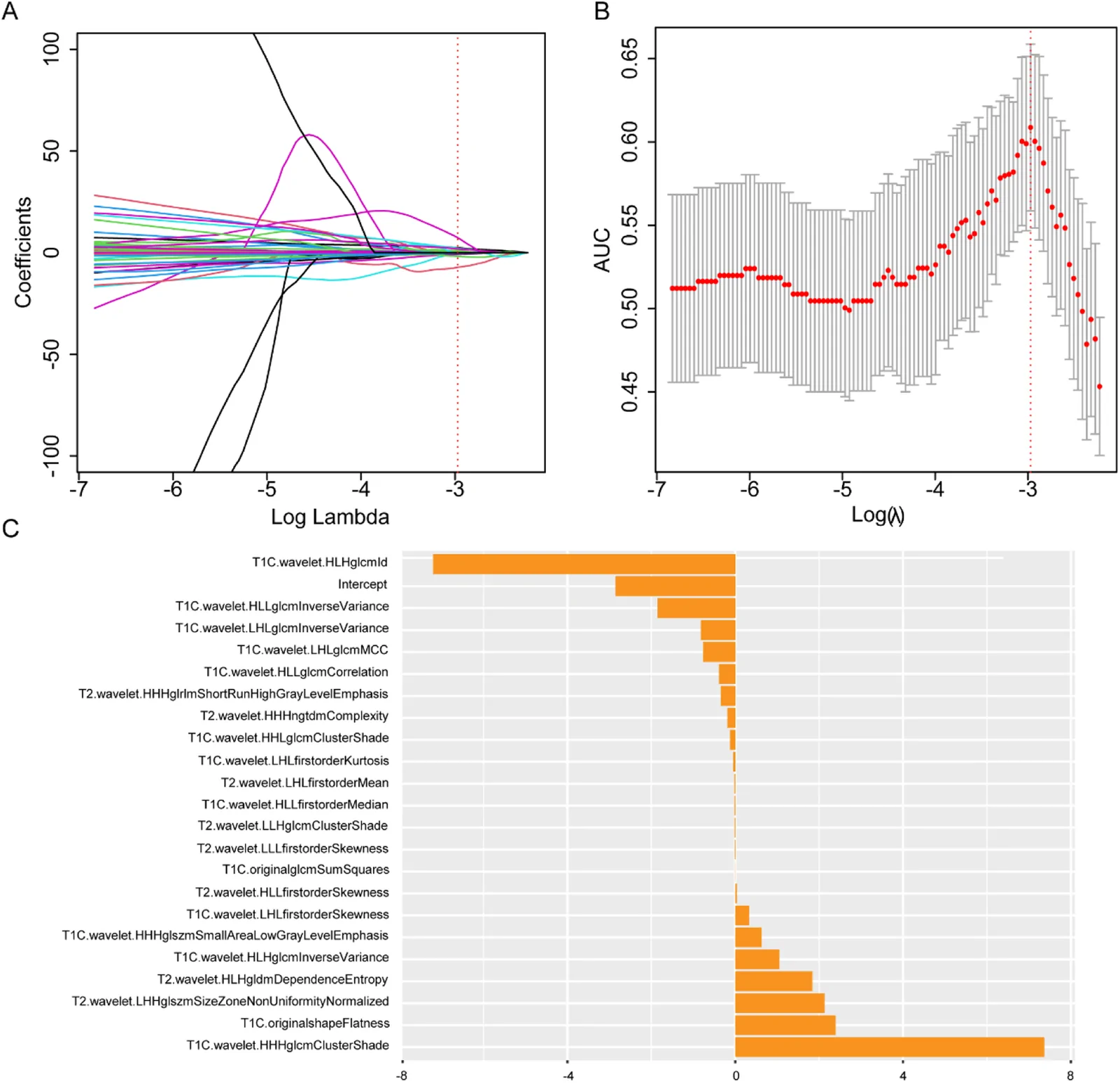
Use of lasso logistic regression to select features. (A) Various features are influenced by changes in regularization parameters (λ). (B) Tenfold cross-validation used to determine the optimal λ for radiomics model construction. The dotted vertical lines indicate the optimal values of the LASSO tuning parameter, and the λ corresponding to the maximum mean AUC in cross-validation (λ_min) was selected (λ = 0.052). (C) 22 features were selected.

### Construction and calibration of the radiomics nomogram

The radiomics nomogram, developed via multivariable logistic regression incorporating T2WI signal intensity (p < 0.001), pseudocapsule (p = 0.001), and Rad-score (p = 0.001), is visually represented in Fig. 3A. Calibration curves (Fig. 3B) demonstrated excellent agreement between predicted probabilities and actual outcomes in the training cohort (p = 0.873), internal validation cohort (p = 0.983), and external validation cohort (p = 0.113). The Receiver Operating Characteristic (ROC) curves of the nomogram in each dataset are shown in Fig. 3C, with diagnostic performance metrics (accuracy, sensitivity, specificity) detailed in Supplementary Figure 3.

**Fig. 3:**
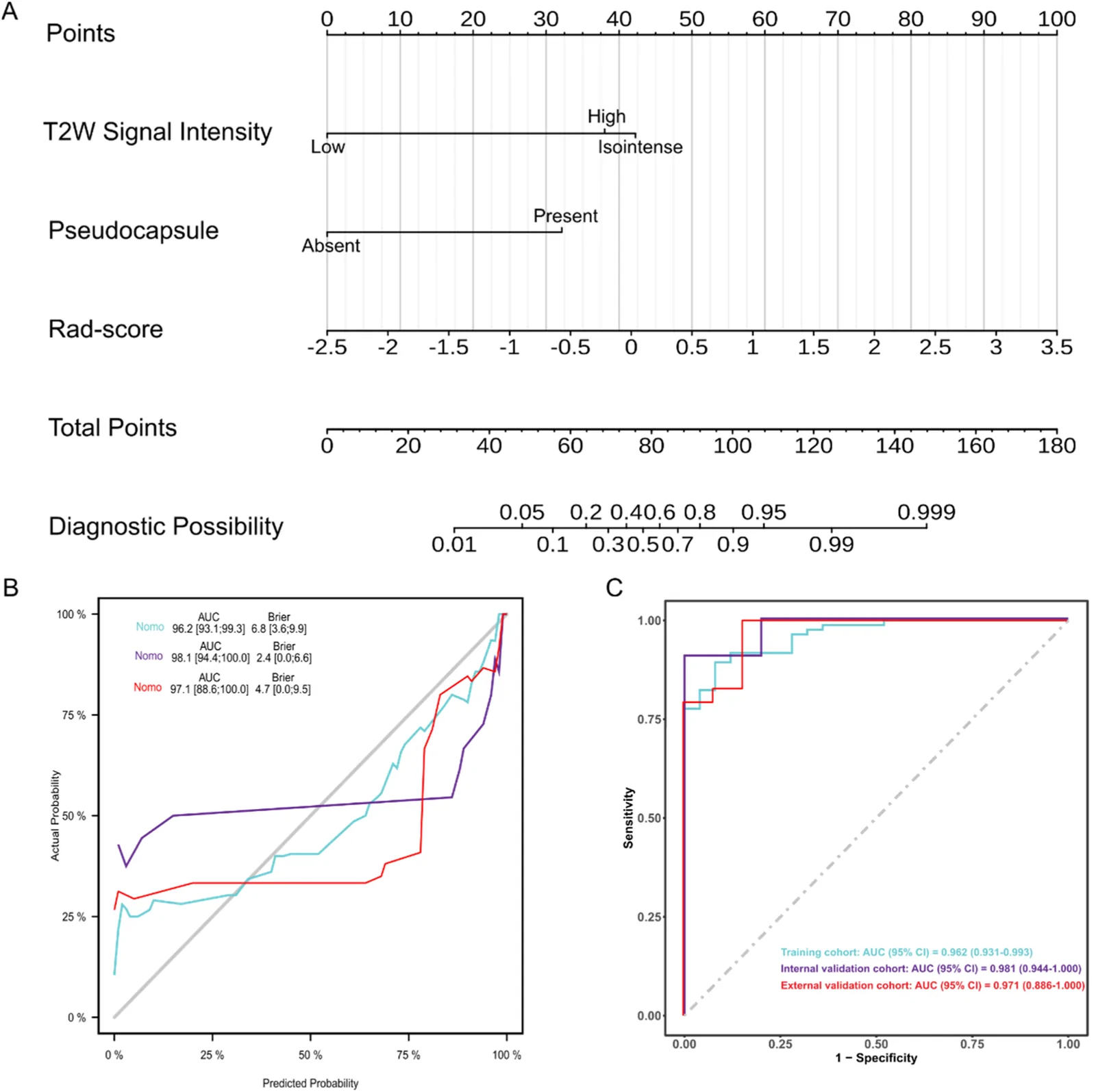
(A) Nomogram integrating T2W signal intensity, pseudocapsule status, and Rad-score to predict SRM malignancy. Points for each feature sum to estimate diagnostic probability. (B) Calibration curves show agreement between predicted and actual malignancy across training (teal), internal validation (purple), and external validation (red) cohorts. Lower Brier scores (training: 0.038; internal: 0.024; external: 0.017) indicate robust calibration. (C) ROC curves demonstrate the nomogram’s discriminative performance: training AUC = 0.962 (95% CI: 0.931–0.993), internal validation AUC = 0.981 (95% CI 0.944–1.000), external validation AUC = 0.971 (95% CI 0.886–1.000).

### Clinical model analysis and multicollinearity assessment

Univariate and multivariate analyses for the clinical model are summarized in Supplementary Table 3. Variance Inflation Factor (VIF) analysis (Supplementary Fig. 4) indicated no significant multicollinearity (all VIF < 5), ensuring model stability.

#### Comparison of model performance

Decision Curve Analysis (DCA) curves for the three models across cohorts are displayed in Fig. 4 (A‒C for different datasets). Additionally, the diagnostic performance metrics of the radiomics nomogram at clinically relevant threshold probabilities are summarized in Supplementary Table 4. Comparative ROC curves between the nomogram, radiomics model, and clinical model are shown in Fig. 5 (A‒C for training, internal validation, and external validation cohorts, respectively), with DCA comparisons as insets. In the training cohort, the nomogram achieved the highest AUC of 0.962 (95% CI 0.931–0.993), outperforming the radiomics model 0.918 (95% CI 0.862–0.974) and clinical model 0.856 (95% CI 0.780–0.933). DeLong tests confirmed significant differences between the nomogram and the clinical model (p = 0.031) or radiomics model (p = 0.037), with no significance between the latter two (p = 0.488). Consistent trends were observed in the internal validation cohort (nomogram: 0.981, 95% CI 0.944–1.000; radiomics model: 0.912, 95% CI 0.750–1.000; clinical model: 0.844, 95% CI 0.578–1.000) and external validation cohort (nomogram: 0.971, 95% CI 0.886–1.000; radiomics model: 0.928, 95% CI: 0.841–0.989; clinical model: 0.916, 95% CI: 0.760–0.976), though statistical significance was not reached (all p > 0.05) likely due to limited sample sizes. The nomogram consistently showed the highest net clinical benefit across threshold probabilities. We have further supplemented the comparison between the nomogram and standard radiological interpretation within this 30%–80% threshold range. The results show that the nomogram outperforms standard radiological interpretation by a net benefit margin of 0.12–0.18 across all key thresholds in the external validation cohort, which directly reflects its added value in improving clinical decision-making (Supplementary Table 5).

**Fig. 4:**
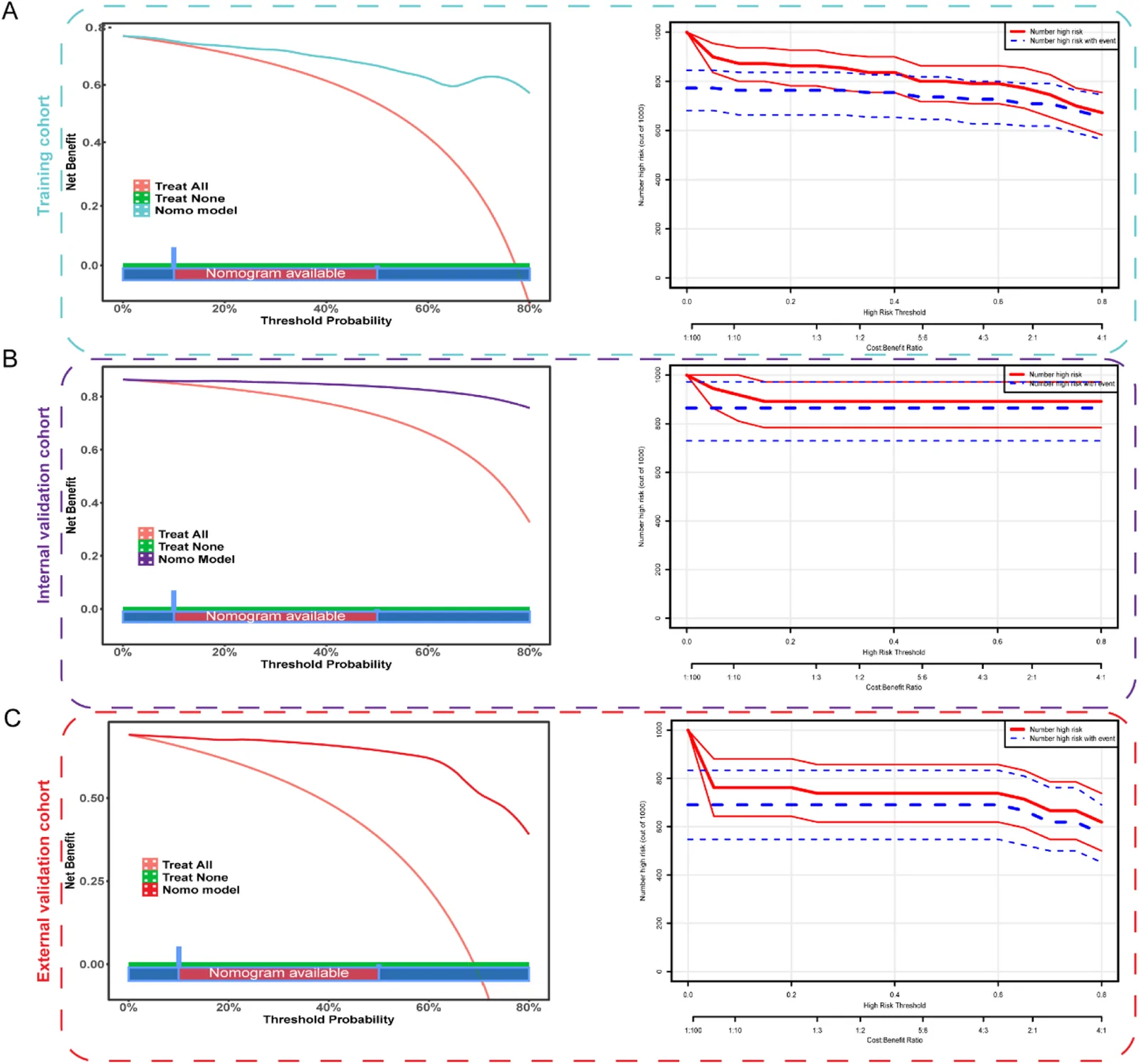
Decision Curve Analysis (DCA) of the radiomics nomogram for Small Renal Mass (SRM) malignancy prediction. (A–C) DCA evaluates the net benefit of the radiomics nomogram (Nomo model) versus extreme strategies (Treat All, Treat None) across training (A), internal validation (B), and external validation (C) cohorts. Plots compare net benefit across threshold probabilities of malignancy. For each cohort, the nomogram (colored curves) outperforms Treat All/Treat None across most threshold ranges (horizontal bars, “Nomogram available”), confirming its clinical utility. Right panels show decision curves with cost-benefit ratios, further validating the nomogram’s superiority in guiding SRM management.

**Fig. 5:**
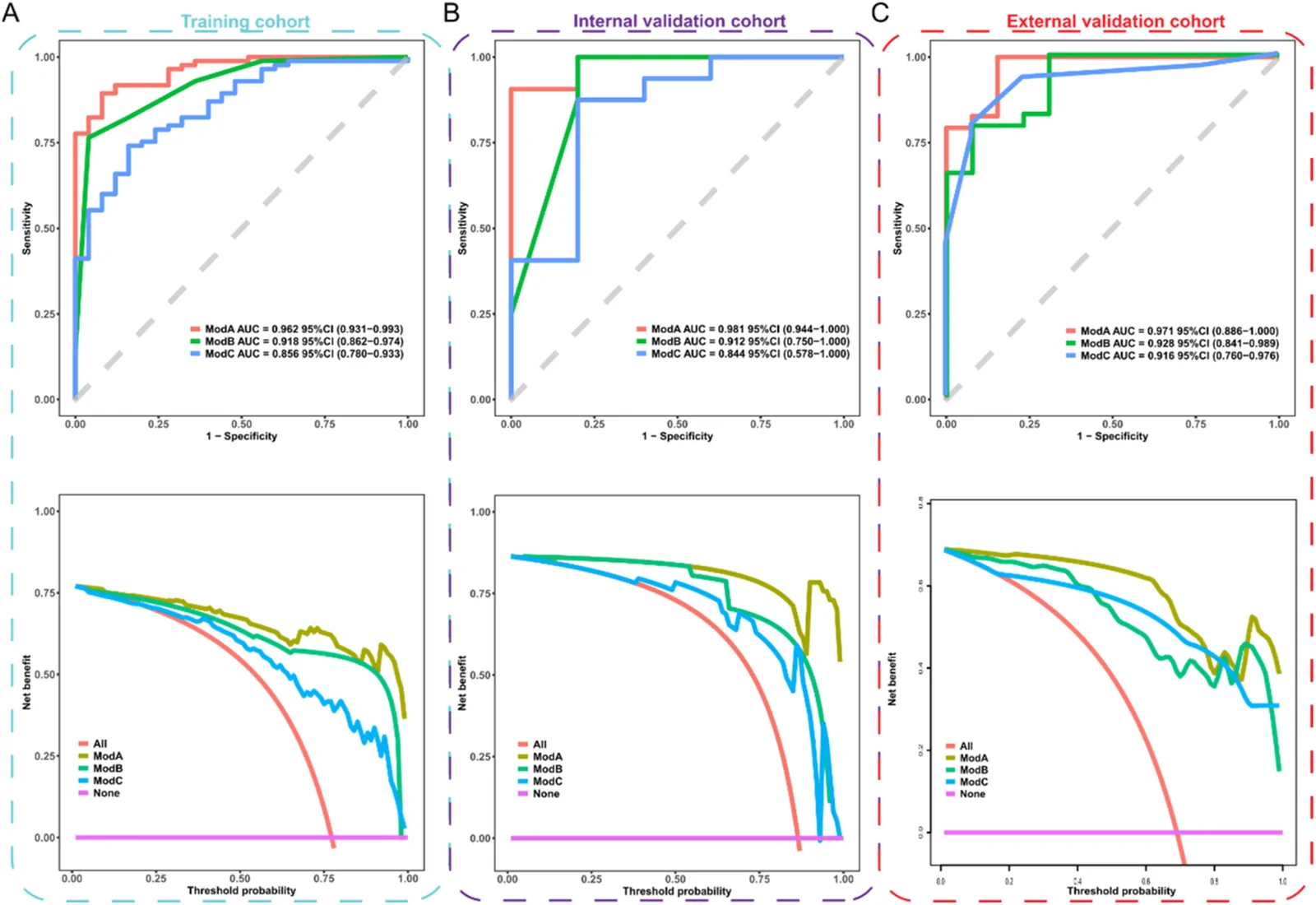
Comparative diagnostic performance of models for Small Renal Mass (SRM) malignancy assessment. (A–C) Receiver Operating Characteristic (ROC) and Decision Curve Analyses (DCA) comparing three models (ModA, ModB, ModC) across training (A), internal validation (B), and external validation (C) cohorts. Top panels show ROC curves, with Area Under the Curve (AUC) and 95% Confidence Intervals (95% CI) indicating discriminative ability (e.g., ModA: training cohort AUC = 0.962, 95% CI 0.931–0.993). Bottom panels present DCA, evaluating net benefit across threshold probabilities of malignancy. The consistent superiority of ModA (red curves) in both ROC (higher AUC) and DCA (greater net benefit vs. All/None strategies) across cohorts confirms its robust performance for SRM malignancy stratification.

## Discussion

In the present study, we developed an integrated diagnostic nomogram that combines conventional MRI features (T2WI signal intensity and pseudocapsule) with MRI-based radiomic features, aiming to preoperatively distinguish benign from malignant Small Renal Masses (SRMs). This model demonstrated robust performance across training, internal validation, and external validation cohorts, confirming its potential as a reliable, non-invasive tool to optimize preoperative decision-making for SRMs. The clinical management of SRMs remains challenging due to the limitations of current diagnostic modalities .^7^ The diagnosis of SRMs primarily relies on imaging examinations such as ultrasound, CT, and MRI .^23^ These imaging modalities are generally preferred because of their safety and noninvasive nature compared with renal mass biopsy. However, some benign small renal tumors, particularly fat-poor Angiomyolipomas (fp-AML) and oncocytomas, often lack distinctive imaging features. These tumors can be challenging to differentiate from renal cell carcinoma, potentially leading to misdiagnosis as malignant tumors and unnecessary surgical interventions .^9^ Our nomogram addresses this gap by integrating quantitative radiomic features with qualitative imaging markers, thereby enhancing diagnostic precision and reducing overtreatment risks.

The selection of T2WI signal intensity and pseudocapsule as key clinical features in our nomogram aligns with existing literature. The pseudocapsule in renal tumors is not a true anatomical capsule. Rather, it forms as the tumor expands outward, compressing the surrounding normal renal tissue, which leads to fibrosis of the compressed renal parenchyma. This results in a dense fibrous band resembling a “capsule”, histologically composed of fibrous connective tissue, inflammatory cells, and atrophic renal tubules .^24^ Joseph M et al. analyzed postoperative pathology from 160 pT1-stage renal tumors, including 60 clear cell carcinomas, 50 papillary renal cell carcinomas, 25 chromophobe renal cell carcinomas, and 25 oncocytomas. They found that pseudocapsule was present in 60 clear cell carcinomas, 47 papillary renal cell carcinomas, 18 chromophobe renal cell carcinomas, and 15 oncocytomas. However, among the oncocytomas, only one case had a complete pseudocapsule, and the pseudocapsule in oncocytomas was the thinnest among all types .^25^ For angiomyolipomas, due to their hamartomata’s nature, there is usually no distinct pseudocapsule between the tumor and normal renal tissue, and they may even show focal invasion into surrounding tissues, this lack of pseudocapsule is a key differential feature from malignant renal tumors .^26^

The T2WI signal intensity of renal lesions is closely related to their histological composition (water content, fat content, proportion of fibrous tissue, and hemorrhage/necrosis etc.). Malignant renal tumors (such as ccRCC) often exhibit high T2WI signal intensity due to cystic degeneration, abundant cytoplasm, and increased interstitial water content. In contrast, benign lesions such as oncocytomas and angiomyolipomas typically show low to moderate T2WI signal intensity: oncocytomas have dense cytoplasm and little interstitial space, while angiomyolipomas contain abundant fat (which shows low signal on T2WI) .^27^ Previous studies have confirmed that T2WI signal intensity is an independent predictor for differentiating malignant from benign renal lesions, with a sensitivity of 78%–85% and a specificity of 72%–80% .^28^ This histological basis explains why T2WI signal intensity is a key component of the clinical model in this study. In the field of clinical models, Canvasser et al. developed a clear cell Likelihood Score (ccLS) to distinguish clear cell Renal Cell Carcinoma (ccRCC) within T1a renal tumor from other types of renal tumor. Their findings demonstrated that when tumors with scores ranging from 4 to 5 were categorized as mpMRI-identified ccRCC, the model achieved an accuracy rate of 79% .^29^ Steinberg et al. conducted a prospective study in which they applied ccLS to preoperatively determine the nature of renal masses across all stages. In the case of SRMs, their study reported an accuracy rate of 74% (95% CI 67–79) .^30^

Compared with conventional imaging modalities, radiomics typically extracts quantitative and ideally replicable information from diagnostic images. At the same time, there are many complex features that are challenging for our human eyes to identify or quantify. This information contributes to clinical decision-making .^31^ Numerous prior studies have employed radiomics based on CT or MRI to determine the nature of renal masses .^28^^,^^32^, ^33^, ^34^, ^35^, ^36^ All the aforementioned studies included renal tumors of all sizes and did not specifically analyze SRMs. However, in clinical practice, it is more challenging to determine the nature of small renal tumors before surgery because the vast majority of benign tumors treated with surgery are small renal tumors. Li et al. reported that a CT-based radiomics nomogram could differentiate SMRs of renal oncocytoma from ccRCC with an AUC of 0.898 in the validation set .^17^ Yang et al. reported that the conventional radiomics CT nomogram outperformed radiomics-only analysis in distinguishing between fp-AML and ccRCC in CT images .^18^ Their analysis was limited to assessing the utility of radiomics in differentiating ccRCC from fp-AML or oncocytoma. To explore the application of radiomics in differentiating the benign and malignant nature of SRMs, Feng et al. enrolled 156 patients with small renal masses (92 malignant and 64 benign) and extracted radiomic features from CT images obtained during the corticomedullary, medullary, and excretory phases. A composite model incorporating age, sex, tumor size, and Radiomics Score (RS) was then constructed. In the training set, the composite model achieved an AUC of 0.988 (95% CI 0.974–1.000), and in the test set, the AUC was 0.968 (95% CI 0.928–1.000)[16] (Supplementary Table 6). Although the study demonstrated promising model performance, it was a single-center retrospective study without an additional external validation set, which raises concerns about potential overfitting.

To further evaluate the clinical utility of our model, we compared the performance of the radiomics nomogram with the widely used ccLS system in the same cohorts (Table 3). In the training cohort, the radiomics nomogram achieved an AUC of 0.962 (95% CI 0.931–0.993), which was higher than that of the ccLS 0.899 (95% CI 0.821–0.976). Similarly, superior performance of the radiomics nomogram was observed in the internal validation cohort (AUC: 0.981 vs. 0.866) and the external test cohort (AUC: 0.971 vs. 0.926). In addition, the radiomics nomogram demonstrated higher overall accuracy and sensitivity across the three cohorts. These findings are consistent with previous reports indicating that although the ccLS system based on contrast-enhanced MRI provides useful diagnostic information, its performance may be limited in certain cases of small renal masses .^37^ By integrating radiomics features with clinical and imaging characteristics, the proposed radiomics nomogram may capture more comprehensive intratumoral heterogeneity, thereby improving the differentiation between benign and malignant small renal masses.

**Table 3:** Comparison of diagnostic performance between the radiomics nomogram and the ccLS across three cohorts.

Model	Training cohort (n = 110)	Internal validation cohort (n = 37)	External test cohort (n = 42)
Radiomics nomogram			
Sensitivity	0.894	1.000	1.000
Specificity	0.920	0.800	0.846
Accuracy	0.900	0.973	0.952
AUC (95% CI)	0.962 (0.931‒0.993)	0.981 (0.944‒1.000)	0.971 (0.886–1.000)
ccLS			
Sensitivity	0.882	0.813	0.724
Specificity	0.800	0.800	1.000
Accuracy	0.864	0.811	0.810
AUC (95% CI)	0.899 (0.821‒0.976)	0.866 (0.602‒1.000)	0.926 (0.867‒0.985)

AUC, Area Under the ROC Curve; CI, Confidence Interval; ccLS, clear cell Likelihood Score.

Our study has several key distinctions from previous studies. Many prior studies have relied on single imaging features or radiomics separately without their integration. In contrast, our model combines conventional imaging features (T2WI signal intensity and pseudocapsule) with multimodal MRI radiomic features, resulting in improved performance. We opted for multimodal MRI images over enhanced CT, as well as a 3D ROI instead of a 2D ROI. These choices allowed us to extract features that represented tumor information more comprehensively and accurately. While previous studies have reported tumor size and sex as independent risk factors for distinguishing between malignant and benign SRMs, this phenomenon was not observed in our study. The superior performance of our integrated nomogram compared to standalone radiomics, or clinical models underscores the value of combining quantitative and qualitative features. Radiomics captures subtle, human-unperceivable patterns (e.g., texture and wavelet features), while conventional MRI features (e.g., pseudocapsule) provide clinically familiar markers that enhance interpretability. This synergy addresses a key limitation of radiomics: its “black box” nature, which can hinder clinician acceptance. By incorporating both, our nomogram balances precision with transparency, making it more actionable in practice. Additionally, our use of MRI over CT offers inherent advantages, including superior soft tissue contrast and avoidance of ionizing radiation, which is particularly relevant for younger patients or those requiring repeated imaging. The 3D ROI segmentation further improves feature reliability by accounting for tumor volume, unlike 2D approaches that may miss critical spatial heterogeneity.

This study has several limitations. First, the retrospective design and relatively small sample size may introduce selection bias. Given that most renal malignancies in this study were clear cell carcinomas and most benign tumors were angiomyolipomas, applying the model to distinguish less common renal tumor types may lead to significant errors. Furthermore, excluding patients without pathological confirmation may have skewed the cohort toward more suspicious lesions, potentially overestimating diagnostic performance. Therefore, future studies with larger sample sizes are needed to balance the distribution across different pathological subtypes of renal tumors. While sensitivity analysis by scanner or center could provide deeper insight into the model’s robustness across different imaging settings, the relative homogeneity of our MRI acquisition protocol helps mitigate potential scanner- or center-related effects. Similarly, although nested cross-validation or bootstrapping could further enhance reliability, our external validation at independent centers has already rigorously assessed the model’s generalization ability in clinical practice. Evaluating the impact of different bin widths could further strengthen our findings; however, sensitivity analysis using alternative bin widths was not performed in this study due to limited manpower. More detailed analyses of these aspects can be undertaken in subsequent larger-scale studies.

Future research should focus on several directions. To clearly evaluate the clinical impact and added value of the proposed nomogram, a prospective multi-reader, multi-case study is necessary in the future. Our prospective study plan is presented in Supplementary Material 11. Incorporating longitudinal data to assess the nomogram’s utility in monitoring indeterminate lesions could further expand its clinical value. Exploring combinations with other modalities (e.g., contrast-enhanced ultrasound or liquid biopsies) may further improve accuracy. Additionally, evaluating the cost-effectiveness of the nomogram is essential to support its widespread clinical adoption.

## Conclusion

The radiomics-clinical nomogram developed in this study exhibits superior performance in preoperatively differentiating benign from malignant Small Renal Masses (SRMs) compared to standalone clinical or radiomics models. Notably, T2WI signal intensity, pseudocapsule, and the Radiomics score (Rad-score) were identified as independent predictive factors for distinguishing the malignant potential of SRMs. This integrated model leverages the complementary strengths of qualitative imaging features and quantitative radiomic analysis, offering a reliable and non-invasive tool to support clinical decision-making, reduce unnecessary surgical interventions, and optimize personalized management of patients with SRMs.

## Ethics approval and consent to participate

This retrospective study was approved by the Institutional Review Boards of both institutions, which waived the requirement for individual informed consent (Ethics approval number: SYSKY-2024-367-01 and B2023-715-Y02). The Registration No. of the study is ChiCTR2500106890.

## Consent for publication

All authors agree to this submission.

## Funding

This work was supported by the 10.13039/501100001809National Natural Science Foundation of China (82303405, 82203720, 82203673), the 10.13039/501100003453Natural Science Foundation of Guangdong Province (2024A1515013201, 2023A1515010355, 2023A1515012486) and the Guangzhou Municipal Basic Research Program Jointly Funded by City, University, and Enterprise Special Project (2024A03J0907).

## Conflicts of interest

The authors declare no conflicts of interest.

## Data Availability

The datasets analyzed during this study are available from the corresponding authors upon reasonable request.
