# Supplementary material for: A multimodal MRI radiomics sonata for differentiating small renal masses: a two-center retrospective study
Source: Clinics (Sao Paulo). 2026 Oct 16;81:101212. doi: 10.1016/j.clinsp.2026.101212 (PMC13691506; doi:10.1016/j.clinsp.2026.101212)
Supplement: Supplementary file 1 [file mmc1.docx]

**CLINICS-D-26-01364_Supplementary Material**

**Supplementary Table 1** Distribution of ICC values for different feature types.

| **Feature Type** | **n** | **ICC (Mean ± SD)** | **ICC (Median [IQR])** | **ICC Range** | **Reproducible n (%)** |
| --- | --- | --- | --- | --- | --- |
| First-order | 36 | 0.96 ± 0.05 | 0.97 [0.97‒0.98] | 0.71‒1.00 | 35/36 (97.2%) |
| Shape | 28 | 0.91 ± 0.12 | 0.98 [0.83‒1] | 0.64‒1.00 | 23/28 (82.1%) |
| Texture | 150 | 0.95 ± 0.12 | 0.98 [0.95‒1] | 0.17‒1.00 | 141/150 (94%) |
| Wavelet | 1488 | 0.97 ± 0.07 | 1.00 [0.97‒1] | 0.03‒1.00 | 1466/1488 (98.5%) |

**Supplementary Table 2** Interpretation of the 22 selected radiomics features.

| **Feature** | **Feature type** | **Image sequence** | **Interpretation** | **Potential biological significance** |
| --- | --- | --- | --- | --- |
| T1C.wavelet.HLHglcmId | Wavelet + GLCM | T1C | Measures local intensity similarity between neighboring voxels | Reflects intratumoral enhancement homogeneity, possibly related to vascular uniformity |
| T1C.wavelet.HLLglcm Inverse Variance | Wavelet + GLCM | T1C | Sensitive to local intensity variation | Indicates micro-scale heterogeneity in contrast enhancement |
| T1C.wavelet. LH Lglcm Inverse Variance | Wavelet + GLCM | T1C | Captures subtle gray-level differences | May reflect heterogeneous vascular permeability or necrosis |
| T1C.wavelet.LHLglcm MCC | Wavelet + GLCM | T1C | Maximal correlation coefficient describing texture complexity | Associated with complex tissue organization |
| T1C.wavelet.HLLglcm Correlation | Wavelet + GLCM | T1C | Measures linear dependency of neighboring voxels | Indicates structured enhancement patterns |
| T2.wavelet.HHHglrlm Short Run High Gray Level Emphasis | Wavelet + GLRLM | T2 | Measures short runs with high intensity | Reflects small hyperintense structures such as edema or cystic components |
| T2.wavelet.HHHngtdm Complexity | Wavelet + NGTDM | T2 | Quantifies texture complexity | Indicates heterogeneous tissue microenvironment |
| T1C.wavelet.HHLglcm Cluster Shade | Wavelet + GLCM | T1C | Measures asymmetry of intensity clusters | Reflects irregular enhancement distribution |
| T1C.wavelet.LHLfirstorder Kurtosis | Wavelet + First-order | T1C | Describes peakedness of intensity distribution | Indicates presence of extreme enhancement values |
| T2.wavelet.LHLfirstorder Mean | Wavelet + First-order | T2 | Average voxel intensity | Reflects overall T2 signal level related to water content |
| T1C.wavelet.HLLfirstorder Median | Wavelet + First-order | T1C | Median signal intensity | Represents central tendency of enhancement |
| T2.wavelet.LLHglcm Cluster Shade | Wavelet + GLCM | T2 | Measures skewness of texture clusters | Indicates asymmetric distribution of T2 intensities |
| T2.wavelet.LLLfirstorder Skewness | Wavelet + First-order | T2 | Measures asymmetry of intensity distribution | Reflects uneven fluid or edema distribution |
| T1C.originalglcm Sum Squares | GLCM | T1C | Variance of gray-level intensities | Reflects overall enhancement variability |
| T2.wavelet.HLLfirstorder Skewness | Wavelet + First-order | T2 | Describes asymmetry of voxel intensity distribution | May indicate heterogeneous edema |
| T1C.wavelet.LHLfirstorder Skewness | Wavelet + First-order | T1C | Measures asymmetry of enhancement intensity | Reflects uneven contrast uptake |
| T1C.wavelet.HHHglszm Small Area Low Gray Level Emphasis | Wavelet + GLSZM | T1C | Detects small low-intensity zones | May correspond to small necrotic or hypovascular regions |
| T1C.wavelet.HLHglcm Inverse Variance | Wavelet + GLCM | T1C | Sensitive to subtle gray-level variations | Reflects fine-scale enhancement heterogeneity |
| T2.wavelet.HLHgldm Dependence Entropy | Wavelet + GLDM | T2 | Measures randomness of voxel dependencies | Indicates structural complexity of tumor tissue |
| T2.wavelet.LHHglszm Size Zone Non Uniformity Normalized | Wavelet + GLSZM | T2 | Quantifies variability of homogeneous zones | Reflects heterogeneity in edema or tumor architecture |
| T1C.originalshape Flatness | Shape | T1C | Ratio describing tumor flatness | Reflects geometric growth pattern |
| T1C.wavelet.HHHglcm Cluster Shade | Wavelet + GLCM | T1C | Measures skewness of texture clusters | Indicates asymmetric contrast enhancement |

**Supplementary Table 3** Univariate and multivariate logistic regression analysis of the clinical factors in training cohort.

| **Factors** | **Univariate analysis** | | | **Multivariate analysis** | | |
| --- | --- | --- | --- | --- | --- | --- |
|  | **OR** | **95%CI** | **p-value** | **OR** | **95%CI** | **p-value** |
| Age (years) | 1.02 | 0.99‒1.06 | 0.185 |  |  |  |
| Gender (Female vs. Male) | 0.20 | 0.07‒0.51 | <0.001 |  |  |  |
| Location (Left vs. Right) | 0.89 | 0.36‒2.19 | 0.801 |  |  |  |
| Tumor Size (cm) | 1.02 | 0.59‒1.76 | 0.952 |  |  |  |
| T2WI Signal Intensity |  |  |  |  |  |  |
| Isointense vs. Low | 15.11 | 3.60‒84.13 | <0.001 | 38.36 | 4.73–310.85 | <0.001 |
| High vs. Low | 27.56 | 8.38‒106.40 | <0.001 | 37.99 | 6.83–211.26 | <0.001 |
| T2WI Signal Heterogeneity |  |  |  |  |  |  |
| Minimally heterogeneous vs. Homogeneous | 2.13 | 0.79‒5.84 | 0.138 |  |  |  |
| Heterogeneous vs. Homogeneous | 26.25 | 4.59–499.20 | <0.001 |  |  |  |
| Pseudocapsule (Present vs. Absent) | 14.78 | 5.05‒43.25 | <0.001 | 23.76 | 4.82–117.176 | <0.001 |
| Bulk Fat (Absent vs. Present) | 0.00 | 0.00‒Inf | 0.988 |  |  |  |
| Contrast Enhancement |  |  |  |  |  |  |
| Moderate vs. Low | 1.09 | 0.241–4.95 | 0.909 |  |  |  |
| High vs. Low | 7.56 | 1.80–32.65 | <0.01 |  |  |  |
| Enhancement Heterogeneity |  |  |  |  |  |  |
| Minimally heterogeneous vs. Homogeneous | 4.28 | 1.41–13.67 | <0.05 |  |  |  |
| Heterogeneous vs. Homogeneous | 12.53 | 1.81‒7.15 | <0.001 |  |  |  |

OR, Odd Ratio; CI, Confidence Interval.

**Supplementary Table 4** Diagnostic metrics of the radiomics nomogram at clinically meaningful thresholds.

| **Cohort** | **Threshold Probability** | **Sensitivity (%) [95% CI]** | **Specificity (%) [95% CI]** | **Net Benefit (at management-altering threshold)** |
| --- | --- | --- | --- | --- |
| Training Cohort | 20% | 89.2 [82.5–94.1] | 78.4 [70.1–85.2] | ‒ |
|  | 40% (management-altering threshold) | 78.5 [70.3–85.3] | 89.1 [82.7–93.8] | ~0.4 |
| Internal Validation Cohort | 20% | 87.6 [79.3–93.5] | 76.2 [67.1–83.8] | ‒ |
|  | 40% (management-altering threshold) | 76.8 [67.7–84.2] | 87.3 [80.1–92.6] | ~0.5 |
| External Validation Cohort | 20% | 85.3 [76.1–91.9] | 74.5 [64.3–82.8] | ‒ |
|  | 40% (management-altering threshold) | 74.2 [64.0–82.5] | 85.7 [77.8–91.5] | ~0.3 |

1. 95% CI, 95% Confidence Interval.

2. The 40% threshold is defined as the management-altering point, guiding the shift from observational follow-up to surgical evaluation.

3. Net benefit values are derived from Decision Curve Analysis (DCA) (Fig. 4).

**Supplementary Table 5** Nomogram net benefit at clinically relevant thresholds within 30%–80% probability range.

| **Threshold Probability (%)** | **Cohort** | **Nomogram Net Benefit** | **Standard Radiological Interpretation Net Benefit** | **Net Benefit Margin (Nomogram vs. Standard Interpretation)** |
| --- | --- | --- | --- | --- |
| 30 | Training | 0.42 | 0.28 | 0.14 |
| 30 | Internal Validation | 0.45 | 0.30 | 0.15 |
| 30 | External Validation | 0.38 | 0.26 | 0.12 |
| 40 | Training | 0.40 | 0.27 | 0.13 |
| 40 | Internal Validation | 0.49 | 0.29 | 0.20 |
| 40 | External Validation | 0.31 | 0.23 | 0.08 |
| 50 | Training | 0.38 | 0.25 | 0.13 |
| 50 | Internal Validation | 0.46 | 0.28 | 0.18 |
| 50 | External Validation | 0.30 | 0.22 | 0.08 |
| 70 | Training | 0.32 | 0.21 | 0.11 |
| 70 | Internal Validation | 0.39 | 0.24 | 0.15 |
| 70 | External Validation | 0.27 | 0.19 | 0.08 |
| 80 | Training | 0.25 | 0.18 | 0.07 |
| 80 | Internal Validation | 0.30 | 0.20 | 0.10 |
| 80 | External Validation | 0.22 | 0.16 | 0.06 |

**Supplementary Table 6** Comparison of the present study.

| **Author** | **Study subjects** | **n** | **Number of centers** | **Model** | **AUC** | **Accuracy** | **Specificity** | **Sensitivity** |
| --- | --- | --- | --- | --- | --- | --- | --- | --- |
| Feng | All pathological types of small renal tumors | 156 | 1 | A composite model incorporating age, sex, tumor size, and Radiomics Score (RS) | 0.968 | 88.6% | 89.3% | 90.0% |
| Ruimeng Yang | Lipid-poor angiomyolipoma vs. clear cell renal cell carcinoma | 163 | 1 | A deep learning model based on corticomedullary phase images from contrast-enhanced CT | 0.900 | 82.0% | 78.0% | 83.0% |
| Li X | Renal oncocytoma vs. clear cell renal cell carcinoma | 122 | 1 | A deep learning model based on corticomedullary phase images from contrast-enhanced CT | 0.898 | 86.5% | 87.0% | 86.5% |

**Supplementary Figure 1**

**Supplementary Figure 2**

**Supplementary Figure 3**

**Supplementary Figure 4**

**Supplementary Material 11** Prospective validation research protocol.

**1. Study Design & Registration**

Design: A multicenter, prospective, diagnostic accuracy study.

Registration: Registration at ClinicalTrials.gov or the Chinese Clinical Trial Registry is required prior to the initiation of the study.

Guidelines: This protocol adheres to the STARD 2015 (Standards for Reporting Diagnostic Accuracy) and TRIPOD (Transparent Reporting of a multivariable prediction model for Individual Prognosis Or Diagnosis) statements.

**2. Objective**

To prospectively validate the diagnostic performance (discrimination and calibration) of a pre-defined, locked MRI-based radiomics nomogram for distinguishing benign from malignant SRMs in a multicenter cohort, and to assess its clinical utility using Decision Curve Analysis (DCA).

**3. Sample Size Calculation (Target: n = 300)**

Assumptions based on retrospective pilot data were as follows: the expected Area Under the Curve (AUC) in the prospective cohort was 0.90, with a null hypothesis AUC of 0.70, representing the boundary of clinical usefulness. The anticipated ratio of malignant to benign lesions was 4:1. The statistical parameters included a type I error (α) of 0.05 (two-sided) and a statistical power (1−β) of 0.90. Using the Obuchowski method for diagnostic accuracy, the minimum required sample size was calculated to be 135 evaluable patients. Considering a potential 10% dropout rate due to poor image quality, motion artifacts, or loss to follow-up, the final target enrollment was set at 150 patients across participating centers.

**4. Participants**

Inclusion Criteria:

• Age ≥18-years.

• Presence of an indeterminate solid or complex cystic renal mass.

• Maximum tumor diameter ≤ 4 cm.

• Scheduled for a dedicated multiparametric renal MRI.

• Planned for surgical resection, biopsy, or active surveillance.

Exclusion Criteria:

• Simple renal cysts (Bosniak I/II).

• Contraindications to MRI (e.g., pacemaker, severe claustrophobia).

• History of renal surgery, ablation, or systemic therapy for renal cancer on the ipsilateral kidney.

• Inability to provide informed consent.

**6. Methodology**

**6.1 MRI Acquisition**

All centers will adhere to a standardized renal MRI protocol, including: Axial T2-Weighted Imaging (T2WI), Diffusion-Weighted Imaging (DWI) with ADC mapping, Multiphasic T1-weighted imaging (pre-contrast, corticomedullary, nephrographic, and delayed phases) using a standardized contrast agent dose and injection rate.

**6.2 Reference Standard (Ground Truth)**

Primary: Histopathology from surgical resection or core-needle biopsy.

Secondary (for benign imaging follow-up): For patients opting for active surveillance, stability or regression on follow-up imaging (minimum 2-years) will be considered as evidence of benignity. A separate sensitivity analysis will be performed excluding these cases.

**6.3 Radiomics Workflow (Model Application)**

Blinding: Radiologists performing segmentation will be blinded to the final pathology.

Segmentation: The entire 3D tumor volume will be semi-automatically segmented on the nephrographic phase and/or T2WI using dedicated software (e.g., 3D Slicer).

Feature Extraction: Features will be extracted using the locked algorithm from our previously developed model (e.g., using PyRadiomics). Features include shape, first-order statistics, and texture matrices (GLCM, GLRLM, GLSZM).

Prediction: The model will output a probability of malignancy for each case.

**7. Statistical Analysis Plan**

**Primary Analysis:**

Discrimination: The primary endpoint is the AUC of the ROC curve. The DeLong test will be used to compare AUCs between subgroups.

Performance Metrics: Accuracy, Sensitivity, Specificity, Positive Predictive Value (PPV), and Negative Predictive Value (NPV) will be calculated at the pre-defined optimal probability threshold.

**Secondary & Supplementary Analysis:**

Clinical Utility: Decision Curve Analysis (DCA) will be performed to quantify the net clinical benefit of using the radiomics model across a range of threshold probabilities, compared to strategies of “treat all” or “treat none”.

Comparison with Radiologists: The model's AUC will be compared to the diagnostic performance (based on ccLS scoring system) of two senior radiologists.

Subgroup Analysis: Performance will be evaluated across different histological subtypes (ccRCC, pRCC, chRCC, oncocytoma) and tumor sizes (≤2 cm vs. 2‒4 cm).
